# Catalytically inactive telomerase in oncogenesis

**DOI:** 10.18632/oncotarget.4404

**Published:** 2015-06-10

**Authors:** Vinay Tergaonkar

**Affiliations:** Institute of Molecular and Cell Biology (A*STAR), Proteos, Singapore

Ends of human chromosomes called telomeres are essential for their structural and functional stability [1]. However, these DNA structures designed to function via a plethora of proteins and RNA molecules that bind them, encounter progressive shortening with each cell division due to the end replication problem. *Terc*, a long non coding (lnc)RNA and the reverse transcriptase TERT which uses *Terc* as primer to extend telomeric DNA are the core components of an enzyme called telomerase which keeps telomere lengths intact in rapidly proliferating stem cells [2]. In contrast, this telomere directed reverse transcriptase activity of telomerase is virtually non-existent in most mammalian somatic cells, as the expression of the TERT subunit is transcriptionally shut off in these cells [3]. However rapidly dividing cells such as those from upto 90% of human cancers rely on telomerase activity to elongate telomeres for proliferation. It is believed that a critical length of telomeres is necessary for capping these structures, preventing genomic instability and subverting cell death or senescence, in cancer cells. Hence transcriptional reactivation of TERT, is critical for reconstitution of telomerase activity and cancer progression [2, 3].

Once reactivated, the telomerase enzyme is believed to primarily regulate oncogenesis via telomere elongation. However, there are several reasons to believe that reactivated TERT and hence reconstituted telomerase do not merely rely on their role on telomeres in promoting oncogenesis. It was shown that alternate lengthening of telomeres (ALT), which can also maintain sufficient telomere lengths in cancer cells, is incapable in functionally replacing telomerase in oncogenic processes [4, 5]. Ectopic expression of TERT in mice (which unlike humans possess long telomeres) leads to enhanced tumor progression, without appreciable effects on telomeres. Most strikingly, lack of telomerase leads to repression of spontaneous tumorigenesis and human cancer cells show dramatic inhibition of growth when hTERT levels are reduced for even a few days, not enough time for a significant shortening of telomeres. Taken together, these and many other similar studies suggest that telomerase can regulate oncogenesis independent of telomeres elongation [4, 5]. Clearly, understanding these roles, evidence for which has been accumulated by various labs and across systems, is of paramount interest.

Several components of the telomerase holoenzyme complex have been implicated in various oncogenic processes including DNA damage response, regulation of p53 and RNA dependent RNA polymerase activity. However, which of these activities mediate key telomere independent roles of telomerase in oncogenesis? Are the roles of these components evolutionarily conserved and are these evident only in context of rapidly dividing cancer cells which have huge demands on their replicative, metabolic and DNA damage response machinery? As a starting point, a precise genetic dissection of the telomerase components to find out which of these impart telomere independent roles in oncogeneis is necessary. Albeit the recognition that oncogenesis is a complex process and it may not be dissected very finely using mouse models, these currently are the best possible models available. Unlike the TERT and *Terc* null mice, which are viable, mice lacking many other telomerase components are either not made or are not viable for easy genetic analyses.

Using TERT and *Terc* null mice both of which lack telomerase activity, thus allowing for dissection of their telomere independent roles, we have found TERT can regulate transcription of genes regulated by oncogenic transcription factors NFκB and Myc [6, 7]. In cancer cells TERT levels are upregulated by enhanced oncogenic signaling via Myc and NFκB, both of which have binding sites on the TERT promoters. In turn TERT, can enhance stability or promoter residence of these factors thereby positively regulating its own levels but in the process also regulating global transcription in cancer cells. May be, this attribute of TERT as a transcriptional regulator is cancer cell specific since continued expression of the TERT promoter and reconstituted telomerase activity is necessary only in these cells. Recent genetic analysis shows that at least in lymphomageneis, TERT mediated oncogenesis is *Terc* independent and hence independent of its telomere function [7]. Indeed this analysis should also be carried out in inflammation (more directly NFκB) driven cancers.

The positive feed forward loop between TERT and transcription factors which bind its promoter, namely Myc and NFκB is key in keeping levels of TERT and hence telomerase high in cancer and the effects of this are seen on cell cycle, proliferation, metabolism and inflammation in cancers with reactivated telomerase activity. Indeed in many experimental settings also, mere overexpression of telomerase has been reported to cause an upregulation of target genes that are direct targets of Myc and NFκB and control these processes, collectively termed as “hallmarks of cancer”. These findings leads one to speculate that the ability of TERT to activate transcription via Myc and NFκB which regulate several hundred target genes controlling most hallmarks of cancer is simply a bi-product of the necessity of cancer cells to regulate TERT expression via these oncogenic transcription factors.

Therapeutic targeting of telomerase has not been successful as most current inhibitors have been designed to target its enzymatic activity. But a more advanced view could be to target the non-canonical activity of TERT, namely its ability to regulate transcription and its own levels, which indirectly will affect the levels and activity of telomerase. It has been previously documented that TERT can also regulates Wnt target genes. Given that extensive cross talk exists between Myc, Wnt and NFκB pathways, therapeutic targeting would dictate deciphering the most direct targets of TERT regulation.
